# 2018 International Consensus Statement on Golf and Health to guide action by people, policymakers and the golf industry

**DOI:** 10.1136/bjsports-2018-099509

**Published:** 2018-09-23

**Authors:** Andrew D Murray, Daryll Archibald, Iain Robert Murray, Roger A Hawkes, Charlie Foster, Kevin Barker, Paul Kelly, Liz Grant, Nanette Mutrie

**Affiliations:** 1 Physical Activity for Health Research Centre, University of Edinburgh, Edinburgh, UK; 2 Sports and Exercise Medicine, University of Edinburgh, Edinburgh, UK; 3 Scottish Collaboration for Public Health Research and Policy, Edinburgh, Scotland; 4 School of Psychology and Public Health, La Trobe University, Melbourne, Victoria, Australia; 5 Department of Trauma and Orthopaedics, University of Edinburgh, Edinburgh, UK; 6 Medical Services, European Tour Golf, Virginia Water, UK; 7 World Golf Foundation, St Augustine, Florida, USA; 8 Centre for Exercise, Nutrition and Health Sciences, University of Bristol, Bristol, UK; 9 International Society of Physical Activity for Health, London, UK; 10 Golf Development, The R&A, St Andrews, UK; 11 Global Health Academy and Usher Institute, University of Edinburgh, Edinburgh, UK

**Keywords:** golf, public health, physical activity, sport, consensus

## Abstract

Scientific and public interest relating to golf and health has increased recently. Players, potential players, the golf industry and facilities, and decision makers will benefit from a better understanding of how to realise potential health benefits and minimise health issues related to golf. We outline an International Consensus on Golf and Health. A systematic literature review informed the development of a survey. Utilising modified Delphi methods, an expert panel of 25 persons including public health and golf industry leaders, took part in serial surveys providing feedback on suggested items, and proposing new items. Predefined criteria for agreement determined whether each item was included within each survey round and in the final consensus. The working group identiﬁed 79 scientifically supportable statement items from literature review and discussions. Twenty-five experts (100%) completed all three rounds of surveys, rating each item, and suggesting modifications and/or new items for inclusion in subsequent surveys. After three rounds, 83 items achieved consensus with each with >75% agreement and <10% disagreement. These items are included in the final International Consensus on Golf and Health. The final consensus presented here can inform scientific knowledge, and action plans for (1) golfers and potential golfers, (2) golf facilities and the golf industry, and (3) policy and decision makers external to golf. These outputs, if widely adopted, will contribute to an improved understanding of golf and health, and aid these groups in making evidence-informed decisions to improve health and well-being.

## Introduction

Recent consensus statements related to sport and health have provided comprehensive, evidence-informed summaries of key issues^1–3^ to help people make informed decisions, and to guide implementation.^4^

Golf is a sport played by over 60 million people on six continents.^5 6^ There has been a recent increase in scientific and public interest relating to golf and health with a decade on decade increase in scientific papers and their uptake.^7 8^ Our 2016 systematically conducted scoping review^7^ and others^9^ have highlighted that golf can provide moderate-intensity physical activity, and may be associated with longevity,^10^ physical health^11 12^ and wellness benefits.^13 14^ Conversely, negative health outcomes including injury^15 16^ and an increased risk of skin cancer^17^ have been associated with playing golf.

The need for a comprehensive, evidence-informed consensus summary of key issues, and key actions with regard to golf and health was recognised by the World Golf Foundation and its constituent members who are golf’s global leaders.

The objectives of this study are:

- To engage leaders at the intersection of health, sport, policy and golf to build a cross-sectoral agreement relating to golf and health.
- To achieve consensus on (1) the health risks and benefits associated with golf, (2) how individuals and populations can improve their health through playing golf or spectating at events, (3) how the golf industry and (4) policymakers can increase opportunities for gaining health benefits through golf and minimise the health risks of golf.

This consensus will enable players, potential players and spectators to benefit from knowledge of how to realise health benefits, and minimise associated health risks related to golf. It will facilitate policymakers to raise awareness and support potential public health interventions, and the golf industry to support education and best practice.

## Methods

The consensus was reached by use of the Delphi method. This is a well-accepted, rigorous and systematic method for achieving consensus of opinion among experts and identifying priorities on real-world issues.^18^ These methods can assist in drawing on the best available evidence, and the opinions and experiences of individuals and the organisations they represent. Methods developed by Dalkey and Helmer^19^ have been refined and adapted for a range of settings including healthcare, sport and policy.^20–27^ The Appraisal of Guidelines for Research and Evaluation 2^28^ instrument was used to inform the conduct of this study.

### Preliminary work: literature review and framework development

**Figure 1:**
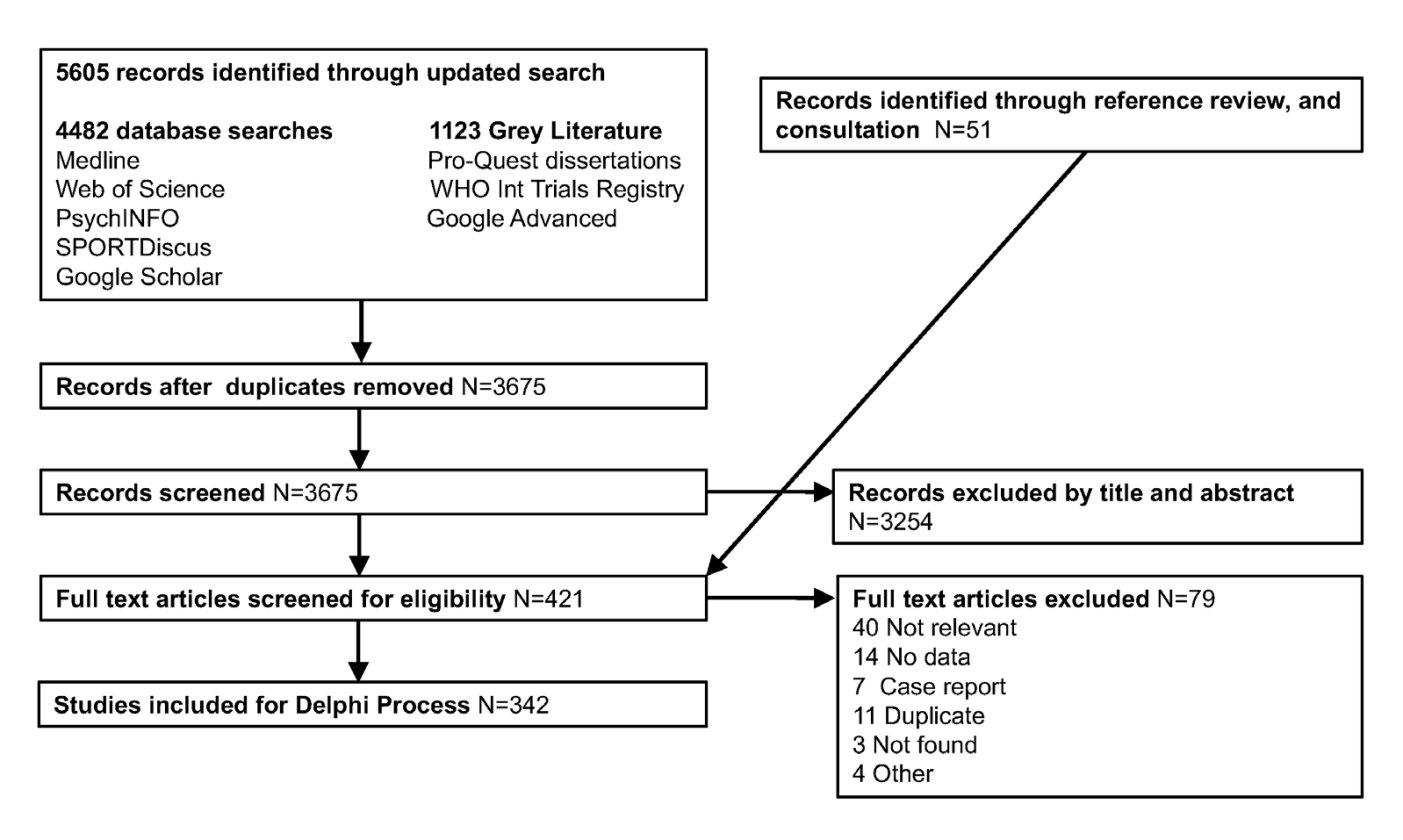
Literature review flow diagram.

A working group of five individuals with expertise in public health, golf and health, policy, industry and research methods was established to facilitate the Delphi consensus process. Preliminary work was conducted by the working group who updated a 2016 systematic search (screening a further 669 relevant records), and extracting further data as shown in figure 1.^7 8^ Relevant guidelines and policy documents were reviewed, and discussions with authors of primary studies and reviews, and other leading authorities were conducted where clarification was helpful.

A framework for organising the available evidence for building a golf and health consensus was developed. Each domain/heading was populated with potential items for inclusion in the proposed survey. A draft survey was generated using Survey Monkey (San Mateo, USA), which was assessed for content and form by the working group and three additional researchers with expertise in public health.

### Selection of expert panel

To maximise objectivity in expert panel selection, it was determined to invite all 25 contributors to the 2018 International Conference on Golf and Health, a satellite meeting of the International Society of Physical Activity for Health 2018 conference. These individuals had an expertise in one or more of (1) public health/physical activity for health policy, (2) the golf and health subject area, and (3) the golf industry. Potential expert panel members were sent an email introducing the concept, and providing a participant information leaflet. Consent was gained electronically.

### Rounds of Delphi survey

#### Round 1

An initial questionnaire with proposed items for the consensus based on the preliminary work of the working group was circulated to the expert panel. Each was invited to grade each item on a five-point Likert scale^29^ (‘strongly agree’, ‘agree’, ‘neither agree nor disagree’, ‘disagree’ and ‘strongly disagree’), and to suggest items and make comments that they thought would add value to the next iteration of the questionnaire. It was stated that the level of evidence for items was variable, and that expert panel input was encouraged. The survey results were collated by the working group.

#### Round 2

The anonymised results from round 1 were fed back to the panel allowing members to appreciate the opinions of others, and the reasons for their position.^26^

Cut-offs for levels of agreement at each round were defined ‘a priori’ following working group discussion. In round 1, items scoring >65% agreement (agree or strongly agree) were included in the questionnaire for round 2. In keeping with established practice, modifications to existing items were incorporated by the working group following review of all expert panel comments from survey 1,^18^ while additional items suggested during round 1 were discussed by the working group and where agreed added to the questionnaire. The 25 original experts were then invited to take part in a second round survey. Participants were invited to rescore each item on the Likert scale, and provide additional comments.

#### Subsequent rounds

Items scoring agreement of >75% in round 2 were included for round 3. Final consensus was defined as items scoring agreement (agree or strongly agree) in 75%,^25 27^ and disagreement (disagree or strongly disagree) in <10% of respondents. The survey process was repeated until consensus had been reached (stability of existing items meeting criteria >85% of items)^24^ and no new items requiring inclusion.

### Data analysis

**Figure 2:**
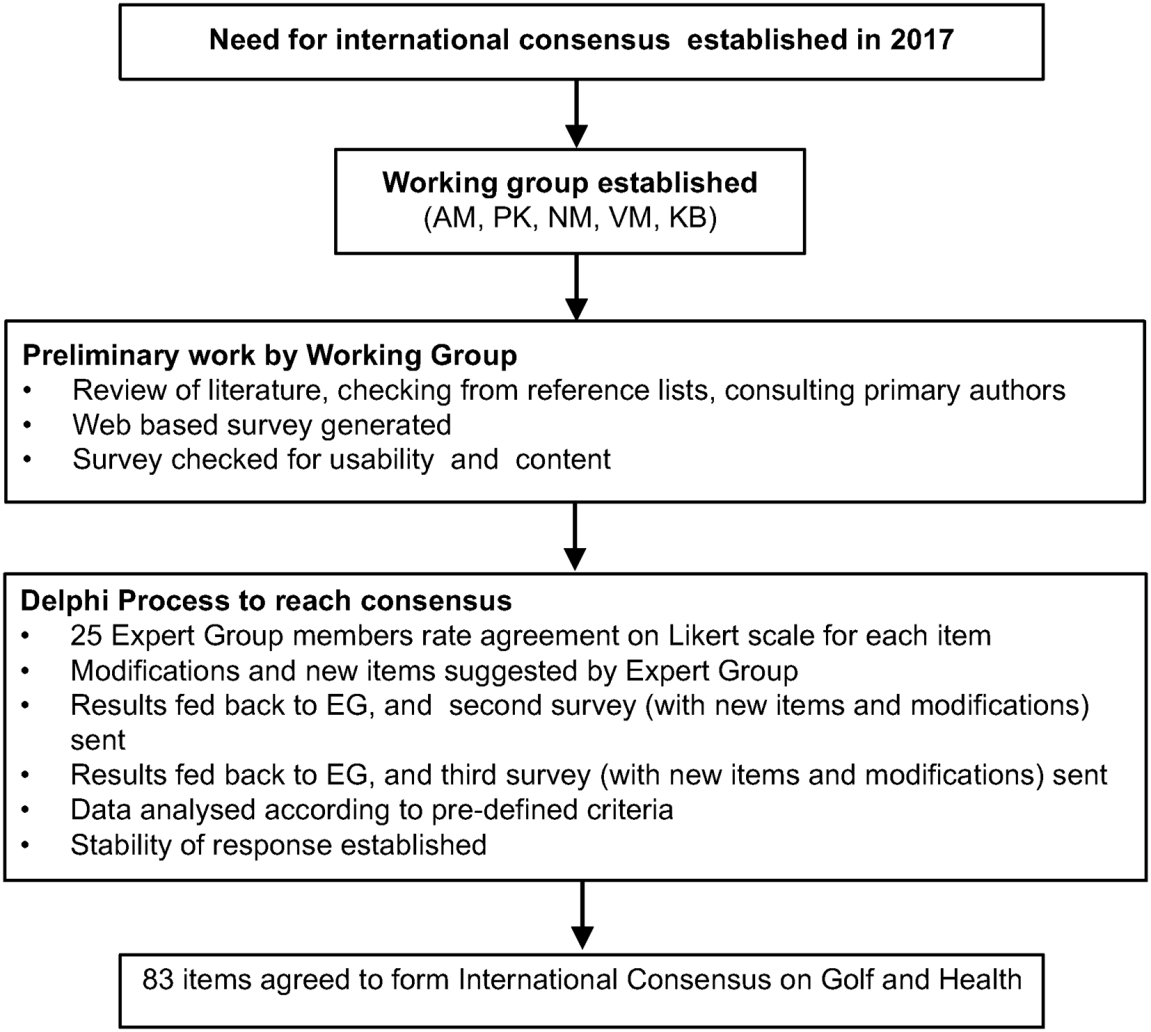
Summary of methods used to develop an International Consensus on Golf and Health. AM, Andrew Murray; EG, expert group; KB, Kevin Barker; NM, Nanette Mutrie; PK, Paul Kelly; VM, Valerie Melvin.

The results of each survey were exported from the Survey Monkey Platform to Excel (Microsoft, Washington, USA). Stacked leaning bar charts (Peltier Tech Advanced V.3.0) were used to present data. A summary of methods is shown in figure 2.

## Results and discussion

### Literature review and framework development

The literature review identified 5605 records. After (1) screening of articles, (2) exclusion of duplicates, (3) further identification of studies through review of references (‘snowballing’) and (4) consultation with subject area experts, 342 articles had data extracted to inform the proposed International Consensus on Golf and Health.

Review of all data sources and working group discussions generated 79 statements/items emerging from the data which were categorised into three broad domains:

- Domain 1: Golf’s associations with health and potential mechanisms.
- Domain 2: Correlates, determinants, diversity and sustainability.
- Domain 3: Interventions and knowledge transfer.

These were further subcategorised as per table 1.

**Table 1:** A framework for building a golf and health consensus

Domain 1: golf’s associations with health and mechanisms			Domain 2: correlates, determinants, diversity and sustainability			Domain 3: interventions/knowledge transfer			
a. Relationship of golf with health outcomes *What are the health benefits/disbenefits of golf?*	b. Mechanisms to achieve health outcomes *How are these benefits developed by golf?*	c. Dose and effect *What is the intensity and/or volume of golf needed for health benefits?*	a. Behavioural patterns *Who plays golf? How much do they play golf?*	b. Correlators and mediators *What helps or hinders participation?*	c. Golf and sustainability *Impact on sustainability/UNSDG*	a. Development and testing *What works to promote golf?*	b. Actions for golfers *How do we maximise health benefits and minimise health risk for golfers?*	c. Actions for golf industry/facilities *What actions can industry/facilities take to benefit health through golf?*	d. Actions for policymakers/decision makers *What actions can policymakers/decision makers take to benefit health through golf?*

UNSDG, United Nations Sustainable Development Goals.

### Establishing consensus using Delphi methods

The results from each round of survey are summarised in table 2. Twenty-five members of the expert group completed each of the three serial surveys within the allocated time frame (a 100% response rate). Following round 1, six new items and 21 modifications were incorporated for survey 2. Following round 2, two new items and 17 modifications were included for round 3. Three iterations or ‘rounds’ of survey were sufficient to collect the required information and reach consensus by predetermined criteria.^18 26^

**Table 2:** Summary of results at completion of each survey round in the Delphi process to establish an International Consensus on Golf and Health

Delphi round	Total number of responses (%)	Total number of items included	Number of survey items progressing to next round	Items modified	New items added
1	25 (100)	79	75	21	6
2	25 (100)	81	81	17	2
3	25 (100)	83	83	0	0

A consensus was considered to have been reached if >75% of experts agreed (‘agree’ or ‘strongly agree’) and <10% indicated disagreement (‘disagree’ or ‘strongly disagree’).

Agreement (defined by >75% ‘agree’/‘strongly agree’ and <10% ‘disagree’/‘strongly disagree’) was achieved for each and all (100%) of 83 individual items included within survey 3. Across all the items, the mean percentage of expert panel that agreed or strongly agreed with statements was >97%.

**Figure 3:**
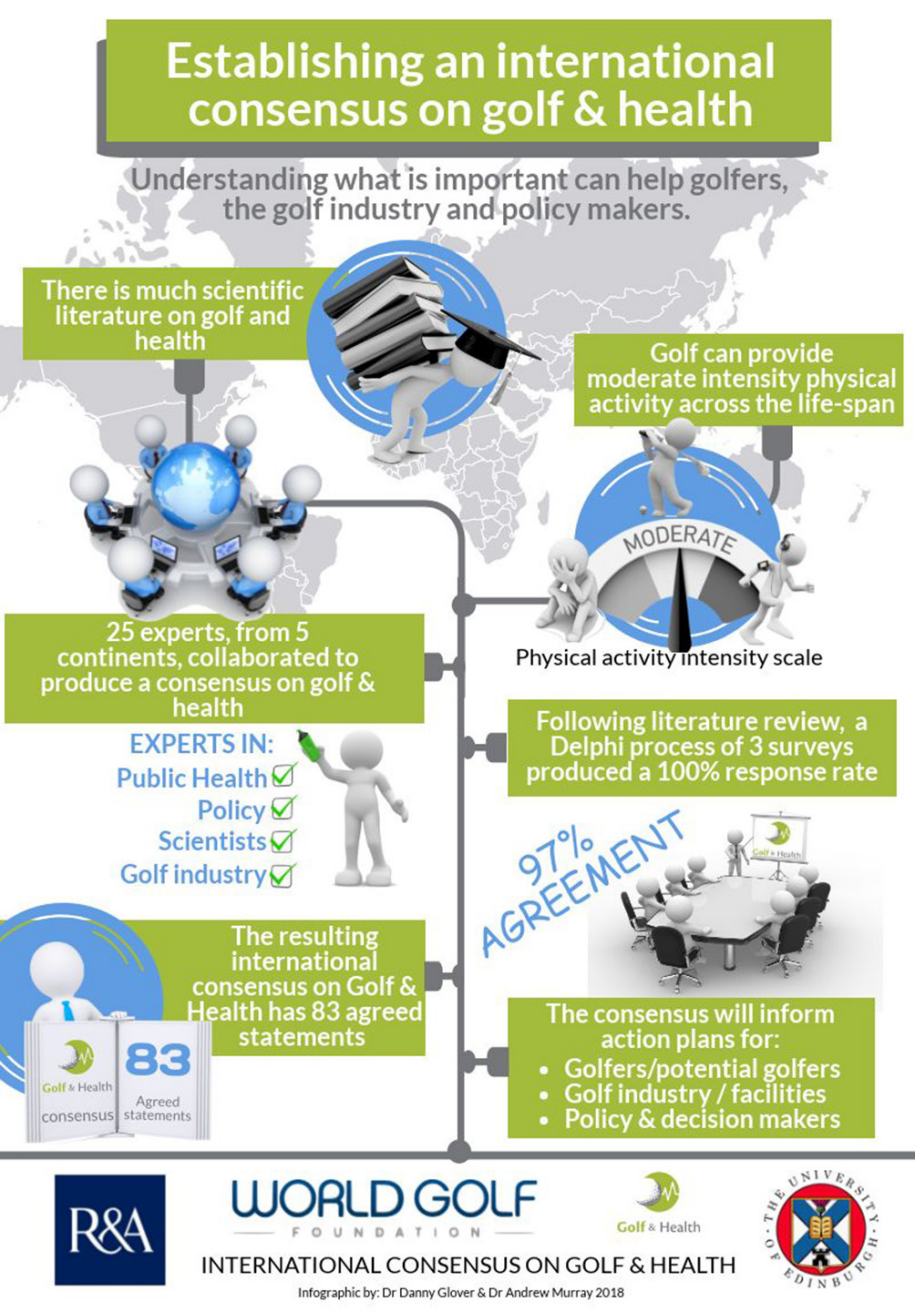
Visual summary of processes establishing an international consensus on golf and health. AM, Andrew Murray; EG, expert group; KB, Kevin Barker; NM, Nanette Mutrie; PK, Paul Kelly; VM, Valerie Melvin.

A summary of processes establishing the consensus are shown in figure 3. All items reaching consensus are shown in table 3, with further detail provided in supplementary file 2.

**Table 3:** Final consensus statements and levels of agreement

Domain 1: golf’s association with health and mechanisms	% Agreement
a. Relationship of golf with health outcomes	
The best available evidence suggests playing golf regularly is associated with increased longevity.	100
Playing golf regularly can improve known risk factors for cardiovascular disease (eg, blood lipids and body composition).	100
As a physical activity, golf is likely to reduce the risk of chronic conditions including cardiovascular disease, type 2 diabetes, colon and breast cancer, depression and dementia.	96
Playing golf is associated with mental well-being benefits which can include improved self-esteem, self-worth, self-efficacy and social connections.	100
Playing/involvement with golf can positively influence health for individuals with disability.	100
Playing golf can contribute to healthy and active ageing, providing physical and mental health, cognitive, social, functional and other benefits.	100
The annual incidence of injury playing golf is moderate compared with other sports, while the risk of injury per hour played is low compared with other sports.	96
Serious injury is rare, although accidental head injury sustained from being struck by a ball or club can have serious consequences.	96
While moderate sun exposure can offer benefits, golfers can be exposed to increased risk of skin cancer associated with excess sun exposure if appropriate care and consideration is not taken.	100
The magnitude of health benefits/health problems will depend on many factors including age, gender, genetic factors and the existing fitness/wellness of the participant, the topography of the course and frequency of play.	100
While a significant body of evidence exists relating to golf and health, further high-quality research is needed.	100
High-quality research is needed to assess relationships between golf and mental health/well-being, the contribution of golf to muscle strength and balance, benefits to particular populations, and to explore cause and effect nature of associations between golf and health.	100
b. Mechanisms to achieve health	
Golf can provide health-enhancing physical activity for persons of all ages.	100
Playing golf can provide moderate-intensity aerobic physical activity.	100
The relative intensity of physical activity while playing golf can vary with topography and length of the course, environmental conditions, and the age, gender and baseline fitness of the participant.	100
Health benefits are likely greater for those walking the course as opposed to riding a golf cart (for those who are able).	100
Benefits accrued by those playing golf riding a golf cart may include health-enhancing physical activity, social connections and green exercise while the intensity of physical activity is lower compared with those playing and walking the course.	92
Playing golf is likely to provide strength and balance benefits for older adults.	100
Spectating in an active fashion (eg, walking the course) at golf courses/tournaments offers an opportunity for health-enhancing physical activity.	100
Playing golf outside can provide a form of green exercise and nature connection which can be enhanced in naturalistic courses.	100
Golf offers opportunities for intergenerational connection, for social interaction and to support communities with events of interest.	100
Taking part in physical activities additional to golf is likely to offer golfers further health benefits.	100
c. Dose and effect	
Adults should do at least 150 min of moderate-intensity aerobic physical activity (which could include golf) throughout the week or do at least 75 min of vigorous-intensity aerobic physical activity throughout the week, or an equivalent combination of moderate and vigorous-intensity activity to meet the WHO recommendations.	100
Participation in golf/other physical activities over and above the minimum physical activity guidelines is likely to offer additional benefits compared with those just reaching the minimum recommendations.	96
Being physically active/playing golf regularly throughout life provides greater benefits than being active/playing golf intermittently.	100

Domain 2: correlates, determinants, diversity and sustainability	% Agreement
a. Behavioural patterns	
Over 20% of adults globally do not meet the WHO Global Recommendations on Physical Activity for Health (WHO figures). Golf is popular in some regions where physical inactivity prevalence is high (North America, Europe, Australasia).	96
Of the over 60 million persons that have played golf at least twice in the previous year, participation is currently highest in North America, Australasia and Europe, in men compared with women, in middle-aged and older adults, in some ethnic groups (White-European heritage) and in those of middle and higher socioeconomic class (The R&A and Sports Marketing Suveys data).	96
b. Correlators and mediators	
There is a need for an inclusive environment within golf that embraces, encourages and welcomes individuals, groups and families from all of society.	100
Some factors that help interest and participation in the sport are that golf can (1) be enjoyable, (2) be played throughout life, (3) offer a sense of community, (4) offer challenge and/or competition, (5) provide outdoor exercise and (6) provide time for self.	96
Golf can also teach life skills, while facilities can provide a social/community hub.	100
Golfers with disability can play equitably with able-bodied golfers or golfers with other types of disabilities at some courses/facilities.	88
Some factors that may hinder interest and participation in the sport include perceptions that it is expensive, less accessible for those from lower socioeconomic groups, male dominated, for older people or difficult to learn.	100
The cost of playing golf can hinder participation in some countries and at some facilities, while other facilities do offer affordable health-enhancing physical activity.	100
Physical proximity to a facility, transport options and playing restrictions can be barriers to participation.	96
Shorter forms of the sport, and efforts to avoid excessively slow play can offset the length of time and offer alternatives to those where time constraints are a barrier to participation.	100
Efforts to provide an infrastructure, social norms and regulations that are welcoming to all can lower barriers to participation.	96
Not everyone will be attracted by the same things at a golf facility, so diversity and specialisation of golf facilities in keeping with the local context, culture and population is appropriate. Aspects that can contribute to people stopping playing golf include: (1) it takes too much time from the family; (2) too expensive; (3) too long to play 18 holes; (4) they tried but did not have fun; (5) too difficult and takes too long to learn; (6) health concerns; and (7) fear of being embarrassed.	88 96
c. Golf and sustainability	
Golf can promote sustainability through practices that support diversity, healthy societies, environmental integrity and prosperity, and well-being at local and global scale.	100

Domain 3: interventions and knowledge transfer	% Agreement
a. Interventions	
Interventions to make the sport more inclusive and welcoming should be supported.	96
More interventions are required to increase access and participation, building on theories around engagement, enjoyment and including effective monitoring and evaluation aspects.	96
The health benefits of golf will be enhanced by appropriate partnership within and outwith the golf sector (eg, with health or education sector organisations).	100
b. Actions for golfers/participants	
Golfers should aim to play golf at least 150 min/week, or engage in other forms of moderate to vigorous physical activities additional to golf.	100
Golfers should be encouraged to walk the course, as opposed to riding a golf cart to obtain optimal health benefits if able.	100
Golfers should be encouraged to make others feel welcome, and support others to enjoy golf.	100
Golfers should warm up with some aerobic exercise, then golf-specific mobility exercises, then practice swings to maximise performance and minimise injury risk.	100
Golfers should be encouraged to maintain hydration (drinking when thirsty and having fluids available) while on the course, particularly in hot and/or humid conditions.	100
Appropriate strength and conditioning exercises can decrease injury and illness risk, and improve performance.	100
Golfers should use sunscreen and appropriate clothing (collared shirt, hat, and so on) as appropriate, and moderate exposure to direct sunlight.	100
Education should be sought regarding playing safely. Children should be adequately supervised.	100
Spectators at golf tournaments can be encouraged to walk, and spectate in an active fashion.	100
Golfers should follow appropriate lightning safety guidelines, and discontinue play if there is danger from lightning.	96
Golf carts when driven should be done so responsibly, and following local guidance including minimum age requirements.	100
Golfers with cardiovascular disease can play with acceptable safety, but should see a doctor should symptoms increase or be unstable.	96
Golfers can be expected to return to golf following total knee, hip or shoulder replacement, with a graduated return to golf.	100
c. Actions for facilities/the golf industry	
Golf facilities and the golf industry should communicate key actions including those generated in this consensus related to golf and health to players, and potential players in a consistent and engaging fashion, appropriate to their context.	100
Grass-roots initiatives supporting development of golf in regions/countries where golf is a relatively new sport can help encourage growth in these areas.	100
Golf facilities and the golf industry should build on existing initiatives promoting inclusivity, and encourage increased participation, by developing environments and price structures that are welcoming to all.	100
Golf facilities and other golf industry leaders and stakeholders should commit and can work together to develop an environment that will inspire and recruit more women and girls to play golf and retain their participation in the game.	96
Golf facilities and the golf industry should encourage effective learning and coaching environments, and support entry-level play, building on existing initiatives.	96
Golf facilities should consider the preferences of the average golfer when setting up the golf course, for example, length of holes and course, depth and nature of rough, severity of hazards, hole positions, and where necessary make adjustments.	80
Facilities should make every effort to promote equality and diversity, and make golf accessible.	100
Golf facilities where possible should consider being multifunctional (having facilities in addition to golf; eg, gym, walking routes or child care) and having diversity of golf facilities.	88
Golf facilities and the golf industry should promote practices that enhance sustainability—maximising opportunities for wildlife conservation, interaction with green space, restricting water, energy and pesticide/chemical use.	100
Golf facilities should be encouraged to provide information and facilities to support golfers warming up to play.	100
The golf industry/golf facilities should encourage players to walk the course if able, and avoid mandatory golf cart use at facilities.	96
The golf industry/golf facilities can encourage and facilitate regular physical activity and other health-enhancing behaviours (eg, healthy eating).	96
The golf industry should educate and protect employees and golfers about the risks of excess sun exposure.	96
Golf facilities should stock sunscreen, hats and collared shirts.	92
Golf facilities and the golf industry should continue to support health and safety regulations, membership of professional organisations, education relating to safe play, and ensure adequate supervision of children.	96
Golf facilities should consider providing cardiopulmonary resuscitation (CPR) training to staff, and provide automatic external defibrillators.	92
Golf carts should be well maintained, with speed limiters and front wheel brakes.	92
Appropriate lightning safety policies and education should be enacted at each facility. Guidance for appropriate action for players should be highlighted by golf facilities and the golf industry.	96
d. Actions for policy/decision makers (outwith golf sector)	
The benefits of regular physical activity including playing golf should be communicated and promoted regularly for persons of all ages, genders and socioeconomic backgrounds.	100
Cross-sectoral policies should be delivered that support the WHO Global Action Plan on Physical Activity, and the United Nations Sustainable Development Goals.	92
Policymakers can be confident golf can provide health-enhancing physical activity to persons of all ages and genders. Policy documents, frameworks and actions should support this.	100
Policymakers should where relevant include golf as a moderate-intensity physical activity in policy documents, guidance and recommendation, and encourage participation for persons of all ages and genders.	100
Policy should support play by diverse geographical and socioeconomic participants of all genders, ages and abilities.	100
Policy documents, frameworks and actions can where relevant usefully acknowledge green space, health and well-being, nature connection, social and community, local and national economic benefits of golf.	96
Policymakers should support efforts to encourage spectators to be physically active (eg, walking the course) at golf and other sporting events.	100
Policies should promote multifunctionality (having facilities in addition to golf) and diversity of facilities where possible, and sustainable practices.	84
Policymakers should work collaboratively with the golf industry and national associations to promote increased participation in physical activity/golf, particularly in groups with low levels of physical activity.	96
Policymakers, governing bodies and the golf industry can work collaboratively to gain acceptance from the International Paralympic Committee that golf be included in the Paralympics.	92

% Agreement is the percentage of expert group members selecting ‘agree’ or ‘strongly agree’.

### Summary of consensus

We aimed to establish a consensus on what is known based on the best available scientific evidence and identified 83 items covering three principle domains by Delphi process. The 25 expert panel members provided representation in global public health and sustainability, physical activity for health, health and sport policy, and included clinicians/academics with golf and health subject knowledge. Senior leaders/accountable officers from the World Golf Foundation, The R&A, the European Disabled Golf Foundation, golf facility managers and professional organisations representing golf coaches internationally provided an industry context vital for the building of consensus, but importantly also for the ongoing engagement of stakeholders able to collaborate and deliver evidence-informed decisions and interventions to improve health and well-being in relation to golf.

Three principal domains were identified within the consensus with critical elements discussed below.

#### Domain 1: golf’s associations with health and mechanisms

This domain included 25 statements, with over 90% of the expert panel agreeing with each item. These statements describe health benefits/disbenefits of golf, the mechanisms by which benefits are achieved, and the volume and intensity of participation needed for these benefits.

##### Relationships of golf with health outcomes

The best available evidence reports golf can have overall health benefits,^7 30 31^ being associated with increased longevity^10 32^ and improving known risk factors for cardiovascular disease.^11 12 33 34^ Golf is associated with mental well-being benefits,^31 35–40^ and can positively influence health for those with disability.^31 41^ Compared with other sports, the annual risk of injury is moderate,^15^ while golfers may be exposed to increased risk of skin cancer.^7 17^ The magnitude of health benefits will depend on many factors including age, gender, genetic factors, and the existing fitness/wellness of the participant, the topography of the course and the frequency of play.^7^ While a significant body of evidence exists relating to golf and health, further high-quality research is needed to assess relationships between golf and mental health, benefits to particular populations, and to explore cause and effect relationships between golf and health.^7 31 42^

##### Mechanisms to achieve health outcomes

Golf can provide social interaction,^38 42–45^ health-enhancing physical activity,^33^ green exercise and nature connection for persons of all ages,^31 42 45 46^ and specifically can provide moderate-intensity aerobic physical activity.^7 33^ Strength and balance benefits are likely for older adults,^47–49^ while further research is needed to assess strength and balance benefits for wider populations.^7^ Health benefits are likely greater for those walking the course as opposed to riding a golf cart, although those playing and riding a cart do gain health benefits.^7 33^ Taking part in physical activities additional to golf is likely to offer further health gains.^50^ Spectating in an active fashion (eg, walking the course) at golf courses/tournaments offers an opportunity for health-enhancing physical activity.^51 52^

##### Dose and effect

Adults should meet WHO recommendations for physical activity.^53 54^ Participation in golf/other physical activities over and above the minimum guidelines is likely to offer additional benefits.^7 50^ Being physically active/playing golf regularly throughout life provides greater benefits than being active/playing golf intermittently.

#### Domain 2: correlates, determinants, diversity and sustainability

This domain included 14 statements that describe who plays golf, what helps or hinders participation, and covers sustainability considerations with respect to golf. Knowledge regarding patterns of participation and determinants is critically important in helping maximise interest and participation in a sport with well-accepted overall health benefits. Golf’s global leadership including The R&A, and the World Golf Foundation have identified challenges related to sustainability including improving diversity of participation, but opportunities to contribute positively and collaboratively towards the United Nations Sustainable Development Goals 2030.^31 46 55 56^

##### Behavioural patterns/participation

Over 60 million people have played golf twice or more in the previous year.^5^ Participation is currently highest in North America, Australasia and Europe, and in in men compared with women, in middle-aged and older adults, in some ethnic groups (White-European heritage) and in those of middle and higher socioeconomic class.^56–59^ Over 20% of adults globally do not meet the WHO Global Recommendations on Physical Activity for Health.^53 60^ Sports programmes that encourage participation across the lifespan have been recognised as an approach that can work to positively impact physical activity.^61 62^

##### Correlators and mediators

To increase participation in sport, there is a need for an inclusive environment that embraces, encourages and welcomes individuals, groups and families from all of society,^31 42 46 62^ and this is true of golf.^31 46 56 57^ Efforts to provide an infrastructure, social norms and regulations that are welcoming to all can lower barriers to participation.^31 46 63^ Some factors that help interest and participation in the sport are that golf can (1) be enjoyable, (2) be played throughout life, (3) offer a sense of community, (4) offer challenge and/or competition, (5) provide outdoor exercise and (6) provide time for self.^31 56 57 63^ Golf can also teach life skills,^45^ while facilities can provide a social/community hub.^31^

Some factors that may hinder interest and participation in the sport include perceptions that it is expensive, less accessible for those from lower socioeconomic groups, male dominated, a sport for older people, or difficult to learn.^31 56 63^ The cost of playing golf can hinder participation in some countries and at some facilities, while other facilities do offer affordable opportunities. Not everyone will be attracted by the same things at a golf facility, so diversity and specialisation of golf facilities in keeping with the local context, culture and population is appropriate.

##### Golf and sustainability

Promoting regular physical activity can support the attainment of a number of the United Nations Sustainable Development Goals.^64^ This consensus recognised the importance of supporting international policy^31 54 55 64^ and best practice in this regard. Golf can work to promote sustainability through practices that prioritise diversity, healthy societies, connection with and care of the environment, environmental integrity, and health and well-being.^31 42 45 46 57^

#### Domain 3: interventions and knowledge transfer

The third domain contains 42 individual items, highlighting its fundamental importance. This section explores what interventions work in promoting golf, and what can practically and feasibly be done to maximise health benefits and minimise health risks associated with golf. The weight of evidence is generally weaker than for other categories, with some recommendations based on consensus of opinion. Practical actions, building on existing progress, can help increase physical activity.^61 62 65^

Included are 13 actions for golfers/potential participants, 18 actions for golf facilities/the golf industry and 10 actions for policy/decision makers external to the golf industry that if widely disseminated and adopted will contribute to an improved understanding of golf and health, and aid these groups in making evidence-informed, more consistent decisions and interventions to improve health and well-being. Representatives from these groups have been key in making these recommendations. These are summarised in the section below, and in table 3. Bite-sized assets (infographics, podcast and video; Murray AD, Infographics and digital resources. An international consensus on gold and health. Under peer review) for golfers, the golf industry and facilities, and policy/decision makers have been produced to facilitate uptake by these groups.

##### Interventions

Appropriate partnerships within, and outwith the sport sector can support interventions to make the sport more inclusive and welcoming.^31 42 45 46 57^ Interventions are required to increase access and participation, building on theories around engagement, enjoyment, and including effective monitoring and evaluation aspects.

##### Actions for golfers/participants

Golfers should aim to play golf at least 150 min/week,^7 53^ or engage in other forms of moderate to vigorous physical activities additional to golf. Golfers can be encouraged to walk the course, as opposed to riding a golf cart if able.^7 66^ Warming up with some aerobic exercise (eg, stair climbing or stationary bike), then golf-specific mobility exercises, then practice swings can help maximise performance and minimise injury risk, as can appropriate strength and conditioning.^67 68^ Golfers should be encouraged to make others feel welcome, and support others to enjoy golf.^31 42^ Spectators at golf tournaments can be encouraged to walk, and spectate in an active fashion.

To minimise health risks, golfers should follow appropriate lightning^69^ and golf cart safety guidelines.^70^ Golfers should use sunscreen and appropriate clothing (collared shirt, hat, and so on) as appropriate,^71^ and moderate exposure to direct sunlight.^72^ Children should be adequately supervised.^7^ Golfers with cardiovascular disease can play with acceptable safety, but should see a doctor should symptoms increase or be unstable.^7^ Golfers can be expected to return to golf following total knee, hip or shoulder replacement, with a graduated return to golf.^73^

##### Actions for golf facilities/the golf industry

Recommendations are presented for golf facilities and the golf industry. The World Golf Foundation and The R&A who lead golf development activity globally are committed to working with a range of stakeholders to deliver and support key actions related to golf and health, and communicate key actions to the 60 million golfers worldwide.

Grass-roots initiatives supporting development of golf in regions/countries where golf is a relatively new sport can help encourage growth in these areas.^6 45 56^ Golf facilities and the golf industry should build on existing initiatives promoting inclusivity, and encourage increased participation by developing environments and price structures that are welcoming to all.^31 42 45 57^ The golf industry/golf facilities can encourage and facilitate regular physical activity, other health-enhancing behaviours (eg, healthy eating), and counsel about the dangers of excessive sun exposure. Practices that enhance sustainability, including maximising opportunities for wildlife conservation, interaction with green space, restricting water, energy and pesticide/chemical use, should be encouraged.^42 56^

Golf facilities and other golf industry leaders and stakeholders can commit and can work together to develop an environment that will inspire and recruit more women and girls to play golf, and retain their participation in the game.^46 56^ Effective learning and coaching environments, and entry-level play, can be further encouraged, with facilities considering the preferences of the average golfer.^63^ Facilities should make every effort to promote equality and diversity, and make golf accessible and environmentally sustainable.^42^ Facilities should consider being multifunctional (having facilities in addition to golf; eg, gym, walking routes or child care) and having diversity of golf facilities.^42^

Further, facilities should be encouraged to:

1. Provide information and facilities to support golfers warming up to play.
2. Stock sunscreen, hats and collared shirts, healthy food and water.^71^
3. Consider providing cardiopulmonary resuscitation training to staff, and provide automatic external defibrillators.^7^
4. Adequately maintain golf carts with speed limiters and front wheel brakes.
5. Provide appropriate lightning safety policies.

##### Actions for policy/decision makers (outwith the golf sector)

Decision makers at community/municipal, local, national and international levels have engaged in discussions which informed this consensus, and future delivery of plans. This consensus has considerable alignment with the WHO Global Action Plan on Physical Activity,^54^ and the United Nations Sustainable Development Goals.^55^ Further cross-sectoral collaboration can further support these global efforts. Policymakers can work collaboratively with the golf industry and national associations/federations to promote increased participation in physical activity/golf, particularly in groups with low levels of physical activity (eg, older adults).

The benefits of regular physical activity including playing golf should be communicated and promoted regularly for persons of all ages, genders and socioeconomic backgrounds. Golf can be included as a moderate-intensity^7 33^ physical activity in policy documents, guidance and recommendations, and participation encouraged for persons of all ages and genders. Policy documents, frameworks and actions can, where relevant, usefully acknowledge green space, health and well-being, nature connection, social and community, and local and national economic benefits of golf.^31^ These policies should support play by diverse geographical and socioeconomic participants, of all genders, ages and abilities, multifunctionality of facilities and sustainability considerations.

### Strengths and limitations of present study

Strengths of the present study include the systematic nature of the literature review, and a 100% response rate from experts identified as leaders across public health/physical activity for health policy, the golf industry, and the golf and health subject area. Recommended standards for the conduct of Delphi studies were followed.^27^ This engagement in coproducing this consensus will aid collaboration in delivering the interventions and action plans that can maximise the impact of this work. We used objective criteria for expert panel selection. The level of agreement for inclusion within the consensus was high, and the threshold for excluding items low, important given the engagement with the golf industry and potential conflict of interest.

Although the search was conducted systematically, using established scoping review methodology,^74 75^ and some quality assessment was carried out, formal and systematic quality assessment of each study was not conducted due to the large range of subjects to be covered. The items are based on the best available evidence, and that in many cases further and more definitive research is needed. Statements contain some element of repetition, which was considered necessary by the working group for the consensus, and action plans by relevant stakeholders to be comprehensive. As evidence and practice evolves, the consensus will require revisiting and updating.

## Conclusion

**Figure 4:**
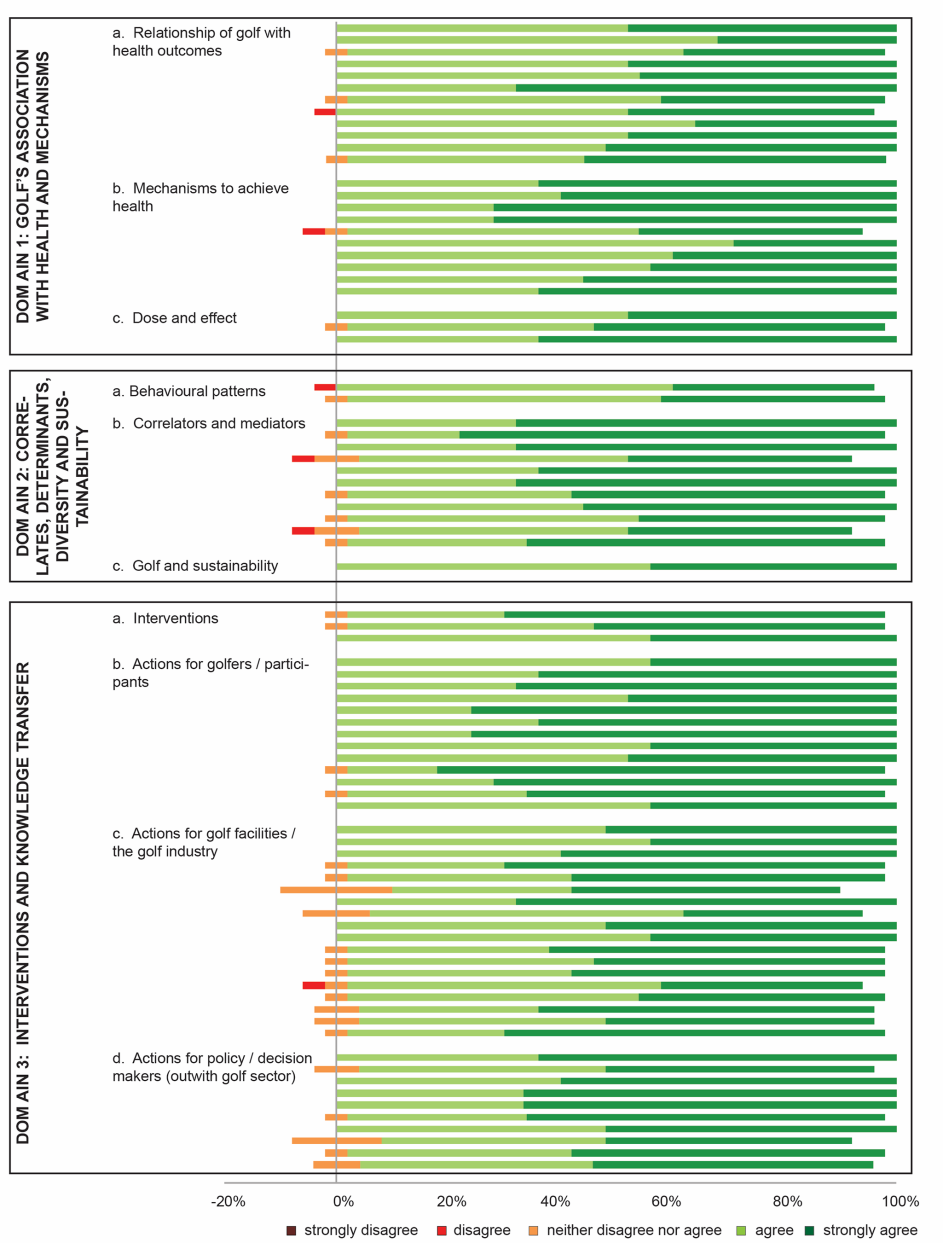
Stacked leaning bar graph showing level of agreement for each item for survey 3.

Our study has produced one of the first wide-ranging global consensus statements for a sport, and engaged leaders at the intersection of health, sport, policy and golf to build this cross-sectoral agreement. Consensus was achieved showing health benefits and health risks that golf is associated with, and highlighting actions by which (1) individuals and populations can improve their health through playing golf, and (2) how the golf industry/facilities and (3) policymakers can increase opportunities to gain health benefits through golf and minimise any health risks associated with golf. These outputs, if widely shared and adopted, will contribute to an improved understanding of golf and health, and aid these groups in making evidence-informed decisions and to improve health and well-being. A stacked leaning bar graph showing level of agreement for each item for survey 3 is shown in figure 4.

10.1136/bjsports-2018-099509.supp1 Supplementary data

10.1136/bjsports-2018-099509.supp2 Supplementary data
