# Supplementary material for: 2018 International Consensus Statement on Golf and Health to guide action by people, policymakers and the golf industry
Source: Br J Sports Med. 2018 Sep 23;52(22):1426–14361. doi: 10.1136/bjsports-2018-099509 (PMC6241627; doi:10.1136/bjsports-2018-099509)
Supplement: Supplementary data [file bjsports-2018-099509supp001.pdf]

Supplementary file 1

Expert Panel members

Dr Daryll Archibald. Lecturer. Public Health. LaTrobe University. Melbourne.

Prof Wade Aubry. Professor of Medicine, and core faculty Institute for Health Policy Studies, University of California.

Tony Bennett. Director. European Disabled Golf Association.

Anthony Blackburn. Founder. Golf in Society, United Kingdom.

Dr Stuart Biddle. Professor of Physical Activity for Health. University of Southern Queensland.

Glenn Cundari. Lead Organiser. 2018 World Scientific Congress of Golf. Technical Director, Professional Golf Association of Canada.

Jackie Davidson. Deputy Director of Golf Development. R&A. St Andrews.

Dr Jose Antonia Doniare. Chief Medical Officer. Royal Spanish Golf Federation. Madrid.

Dr Charlie Foster. President, International Society of Physical Activity for Health.

Prof Liz Grant. Director Global Health Academy and Assistant Principal for Global Health, University of Edinburgh.

Dr Roger Hawkes. Executive Director, Golf and Health. World Golf Foundation, St Augustine, Florida. Member International Golf Federation medical committee.

Dr Tom Hospel. Chief Medical Officer for the Professional Golf Association Tour, and the United States Golf Association.

Dr Prakash Jayabalan. Physician Scientist. Shirley Ryan Ability Lab, and Assistant Professor, North-western University, Chicago.

Val Melvin. International level golf player, and golf industry leader.

Dr Andrew Murray. Consultant in Sports Medicine, University of Edinburgh. Chief Medical Officer, European Tour Golf.

Prof. Nanette Mutrie. Policy Advisor, Scottish Government and Director of Physical Activity for Health Research Centre, University of Edinburgh.

Ian Randell. Chief Executive. Professional Golf Association of Europe.

Dr George Salem. Associate Professor/Director, Anatomical Sciences, University of Southern California.

Dr Kevin Scheepers. Consultant in Sports Medicine and General Practice. Managed Health. Johannesburg.

Dr Dinesh Sirisena. Sports and Exercise Medicine consultant. Khoo Tech Puat Hospital, Singapore.

Jason Stanton. Operations Director. MyTime Active. United Kingdom.

Bradley Stenner. Lecturer. School of Health Sciences. University of South Australia.

Prof Maria Stokes OBE. Professor of Musculoskeletal Rehabilitation, University of Southampton.

Frank Thomas. Founder, Frankly Golf. Former Technical Director, United States Golf Association.

Dr Rehema White. Lecturer, Department of Geography and Sustainable Development. University of St Andrews.
