# Supplementary material for: 2018 International Consensus Statement on Golf and Health to guide action by people, policymakers and the golf industry
Source: Br J Sports Med. 2018 Sep 23;52(22):1426–14361. doi: 10.1136/bjsports-2018-099509 (PMC6241627; doi:10.1136/bjsports-2018-099509)
Supplement: Supplementary data [file bjsports-2018-099509supp002.pdf]

Supplementary File 2

Final consensus statements, and levels of agreement

DOMAIN 1: GOLF'S ASSOCIATIONS WITH HEALTH AND MECHANISMS

a. Relationship of golf with health outcomes

Question No.	Item	Strongly disagree	Disagree	Neither disagree nor agree	Agree	Strongly agree	% Agree/ Strongly Agree
1	The best available evidence suggests playing golf regularly is associated with increased longevity	0.00%	0.00%	0.00%	52.00%	48.00%	**100.00%**
2	Playing golf regularly can improve known risk factors for cardiovascular disease (for example blood lipids, and body composition)	0.00%	0.00%	0.00%	68.00%	32.00%	**100.00%**
3	As a physical activity, golf is likely to reduce the risk of chronic conditions including cardio-vascular disease, type 2 diabetes, colon and breast cancer, depression and dementia	0.00%	0.00%	4.00%	60.00%	36.00%	**96.00%**
4	Playing golf is associated with mental well-being benefits which can include improved self-esteem, self-worth, self-efficacy and social connections.	0.00%	0.00%	0.00%	52.00%	48.00%	**100.00%**
5	Playing/ involvement with golf can positively influence health for individuals with disability	0.00%	0.00%	0.00%	54.17%	45.83%	**100.00%**
6	Playing golf can contribute to healthy and active ageing, providing physical and mental health, cognitive, social, functional and other benefits	0.00%	0.00%	0.00%	32.00%	68.00%	**100.00%**
7	The annual incidence of injury playing golf is moderate compared to other sports , while the risk of injury per hour played is low compared to other sports	0.00%	0.00%	4.00%	56.00%	40.00%	**96.00%**
8	Serious injury is rare, although accidental head injury sustained from being struck by a ball or club can have serious consequences	0.00%	4.00%	0.00%	52.00%	44.00%	**96.00%**
9	While moderate sun exposure can offer benefits, golfers can be exposed to increased risk of skin cancer associated with excess sun exposure if appropriate care and consideration is not taken.	0.00%	0.00%	0.00%	64.00%	36.00%	**100.00%**
10	The magnitude of health benefits/ health problems will depend upon many factors including age, gender, genetic factors and the existing fitness/ wellness of the participant, the topography of the course, and frequency of play.	0.00%	0.00%	0.00%	52.00%	48.00%	**100.00%**
11	While a significant body of evidence exists relating to golf and health, further high quality research is needed	0.00%	0.00%	0.00%	48.00%	52.00%	**100.00%**
12	High quality research is needed to assess relationships between golf and mental health/ well-being, the contribution of golf to muscle strength and balance, benefits to particular populations, and to explore cause and effect nature of associations between golf and health.	0.00%	0.00%	4.00%	44.00%	56.00%	**100.00%**

b. Mechanisms to achieve health

Question No.	Item	Strongly disagree	Disagree	Neither disagree nor agree	Agree	Strongly agree	% Agree/ Strongly Agree
13	Golf can provide health enhancing physical activity for persons of all ages	0.00%	0.00%	0.00%	36.00%	64.00%	**100.00%**
14	Playing golf can provide moderate intensity aerobic physical activity	0.00%	0.00%	0.00%	40.00%	60.00%	**100.00%**
15	The relative intensity of physical activity while playing golf can vary with topography and length of the course, environmental conditions, and the age, gender and baseline fitness of the participant	0.00%	0.00%	0.00%	28.00%	72.00%	**100.00%**
16	Health benefits are likely greater for those walking the course as opposed to riding a golf-cart (for those that are able).	0.00%	0.00%	0.00%	28.00%	72.00%	**100.00%**
17	Benefits accrued by those playing golf riding a golf-cart may include health enhancing physical activity, social connections and green exercise while the intensity of physical activity is lower compared to those playing and walking the course.	0.00%	4.00%	4.00%	52.00%	40.00%	**92.00%**
18	Playing golf is likely to provide strength and balance benefits for older adults	0.00%	0.00%	0.00%	70.83%	29.17%	**100.00%**
19	Spectating in an active fashion (for example walking the course) at golf courses/tournaments offers an opportunity for health enhancing physical activity	0.00%	0.00%	0.00%	60.00%	40.00%	**100.00%**
20	Playing golf outside can provide a form of green exercise and nature connection which can be enhanced in naturalistic courses.	0.00%	0.00%	0.00%	56.00%	44.00%	**100.00%**
21	Golf offers opportunities for intergenerational connection, for social interaction and to support communities with events of interest.	0.00%	0.00%	0.00%	44.00%	56.00%	**100.00%**
22	Taking part in physical activity additonal to golf is likely to offer golfers further health benefits.	0.00%	0.00%	0.00%	36.00%	64.00%	**100.00%**

c. Dose and effect

Question No.	Item	Strongly disagree	Disagree	Neither disagree nor agree	Agree	Strongly agree	% Agree/ Strongly Agree
23	Adults should do at least 150 minutes of moderate-intensity aerobic physical activity (which could include golf) throughout the week or do at least 75 minutes of vigorous-intensity aerobic physical activity throughout the week or an equivalent combination of moderate and vigorous-intensity activity to meet World Health Organisation recommendations.	0.00%	0.00%	0.00%	52.00%	48.00%	**100.00%**
24	Participation in golf/ other physical activities over and above the minimum Physical Activity guidelines, is likely to offer additional benefits compared to those just reaching the minimum recommendations.	0.00%	0.00%	4.00%	44.00%	52.00%	**96.00%**
25	Being physically active/ playing golf regularly throughout life provides greater benefits than being active/ playing golf intermittently	0.00%	0.00%	0.00%	36.00%	64.00%	**100.00%**

DOMAIN 2: CORRELATES, DETERMINANTS, DIVERSITY AND SUSTAINABILITY

a. Behavioural patterns

Question No.	Item	Strongly disagree	Disagree	Neither disagree nor agree	Agree	Strongly agree	% Agree/ Strongly Agree
26	Over 20% of adults globally do not meet the World Health Organisation (WHO) Global Recommendations on Physical Activity for Health (WHO figures). Golf is popular in some regions where physical inactivity prevalence is high (North America, Europe, Australasia).	0.00%	4.00%	0.00%	60.00%	36.00%	**96.00%**
27	Of the over 60 million persons that have played golf at least twice in the previous year, participation is currently highest in North America, Australasia and Europe, in males compared with females, in middle aged and older adults, in some ethnic groups (White-European Heritage) and in those of middle and higher socio-economic class (R&A, and Sports Marketing Suveys Inc. data).	0.00%	0.00%	4.00%	56.00%	40.00%	**96.00%**

b. Correlators and mediators

Question No.	Item	Strongly disagree	Disagree	Neither disagree nor agree	Agree	Strongly agree	% Agree/ Strongly Agree
28	There is a need for an inclusive environment within golf that embraces, encourages and welcomes individuals, groups and families from all of society.	0.00%	0.00%	0.00%	32.00%	68.00%	**100.00%**
29	Some factors that help interest and participation in the sport, are that golf can i) be enjoyable, ii) be played throughout life, iii) offer a sense of community, iv) offer challenge and/ or competition, v) provide outdoor exercise and vi) provide time for self.	0.00%	0.00%	4.00%	20.00%	76.00%	**96.00%**
30	Golf can also teach life skills, while facilities can provide a social/ community hub.	0.00%	0.00%	0.00%	32.00%	68.00%	**100.00%**
31	Golfers with a disability can play equitably with able-bodied golfers or golfers with other types of disabilities at some courses/ facilities.	0.00%	4.00%	8.00%	48.00%	40.00%	**88.00%**
32	Some factors that may hinder interest and participation in the sport, include perceptions that it is expensive, less accessible for those from lower socio-economic groups, male dominated, for older people, or difficult to learn.	0.00%	0.00%	0.00%	36.00%	64.00%	**100.00%**
33	The cost of playing golf can hinder participation in some countries and at some facilities, while other facilities do offer affordable health enhancing physical activity.	0.00%	0.00%	0.00%	32.00%	68.00%	**100.00%**
34	Physical proximity to a facility, transport options and playing restrictions can be barriers to participation.	0.00%	0.00%	4.00%	40.00%	56.00%	**96.00%**
35	Shorter forms of the sport, and efforts to avoid excessively slow play can offset the length of time and offer alternatives to those where time constraints are a barrier to participation.	0.00%	0.00%	0.00%	44.00%	56.00%	**100.00%**
36	Efforts to provide an infrastructure, social norms and regulations that are welcoming to all can lower barriers to participation.	0.00%	0.00%	4.00%	52.00%	44.00%	**96.00%**
37	Not everyone will be attracted by the same things at a golf facility, so diversity and specialisation of golf facilities in keeping with the local context, culture and population is appropriate.	0.00%	4.00%	8.00%	48.00%	40.00%	**88.00%**
38	Some reported aspects that can contribute to people stopping playing golf include i) takes too much time from the family; ii) too expensive; ii) too long to play 18 holes; iv) tried but didn't have fun; v) considered too difficult and takes too long to learn; vi)health concerns and vii) fear of being embarrassed.	0.00%	0.00%	4.00%	32.00%	64.00%	**96.00%**

c. Golf and sustainability

Question No.	Item	Strongly disagree	Disagree	Neither disagree nor agree	Agree	Strongly agree	% Agree/ Strongly Agree
39	Golf can promote sustainability through practices that prioritise diversity, healthy societies, environmental integrity and health and well-being	0.00%	0.00%	0.00%	56.00%	44.00%	**100.00%**

DOMAIN 3: INTERVENTIONS AND KNOWLEDGE TRANSFER

a. Interventions

Question No.	Item	Strongly disagree	Disagree	Neither disagree nor agree	Agree	Strongly agree	% Agree/ Strongly Agree
40	nterventions to make the sport more inclusive, and welcoming should be supported.	0.00%	0.00%	4.00%	28.00%	68.00%	**96.00%**
41	More interventions are required to increase access and participation, building on theories around engagement, enjoyment, and including effective monitoring and evaluation aspects.	0.00%	0.00%	4.00%	44.00%	52.00%	**96.00%**
42	The health benefits of golf can be enhanced by appropriate partnerships within and outwith the golf sector (for example with health or educations sector organisations).	0.00%	0.00%	0.00%	56.00%	44.00%	**100.00%**

b. Actions for golfers / participants

Question No.	Item	Strongly disagree	Disagree	Neither disagree nor agree	Agree	Strongly agree	% Agree/ Strongly Agree
43	Golfers should aim to play golf at least 150 minutes per week, or engage in other forms of moderate to vigorous physical activities additional to golf	0.00%	0.00%	0.00%	56.00%	44.00%	**100.00%**
44	Golfers should be encouraged to walk the course, as opposed to riding a golf cart to obtain optimal health benefits if able.	0.00%	0.00%	0.00%	36.00%	64.00%	**100.00%**
45	Golfers should be encouraged to make others feel welcome, and support others to enjoy golf.	0.00%	0.00%	0.00%	32.00%	68.00%	**100.00%**
46	Golfers should warm up with some aerobic exercise, then golf specific mobility exercises, then practice swings to maximise performance and minimise injury risk.	0.00%	0.00%	0.00%	52.00%	48.00%	**100.00%**
47	Golfers should be encouraged to maintain hydration (drinking when thirsty, and having fluids available) while on the course, particularly in hot and/ or humid conditions.	0.00%	0.00%	0.00%	24.00%	76.00%	**100.00%**
48	Appropriate strength and conditioning exercises can decrease injury and illness risk, and improve performance	0.00%	0.00%	0.00%	36.00%	64.00%	**100.00%**
49	Golfers should utilise sun-screen and appropriate clothing (collared shirt, hat, etc) as appropriate, and moderate exposure to direct sunlight	0.00%	0.00%	0.00%	24.00%	76.00%	**100.00%**
50	Education should be sought regarding playing safely. Children should be adequately supervised.	0.00%	0.00%	0.00%	56.00%	44.00%	**100.00%**
51	Spectators at golf tournaments can be encouraged to walk, and spectate in an active fashion	0.00%	0.00%	0.00%	52.00%	48.00%	**100.00%**
52	Golfers should follow appropriate lightning safety guidelines, and discontinue play if there is danger from lightning.	0.00%	0.00%	4.00%	16.00%	80.00%	**96.00%**
53	Golf carts when driven should be done so responsibly, and following local guidance including minimum age requirements.	0.00%	0.00%	0.00%	28.00%	72.00%	**100.00%**
54	Golfers with cardiovascular disease can play with acceptable safety, but should see a doctor should symptoms increase or be unstable.	0.00%	0.00%	4.00%	32.00%	64.00%	**96.00%**
55	Golfers can be expected to return to golf following total knee, hip, or shoulder replacement, with a graduated return to golf.	0.00%	0.00%	0.00%	56.00%	44.00%	**100.00%**

c. Actions for golf facilities / the golf industry

Question No.	Item	Strongly disagree	Disagree	Neither disagree nor agree	Agree	Strongly agree	% Agree/ Strongly Agree
56	Golf facilities and the golf industry should communicate key actions related to golf and health to players, and potential players in a consistent and engaging fashion, appropriate to their context.	0.00%	0.00%	0.00%	48.00%	52.00%	**100.00%**
57	Grassroots initiatives supporting development of golf in regions/ countries where golf is a relatively new sport can help encourage growth in these areas	0.00%	0.00%	0.00%	56.00%	44.00%	**100.00%**
58	Golf facilities and the golf industry should build on existing initiatives promoting inclusivity, and encourage increased participation by developing environments and price structures that are welcoming to all.	0.00%	0.00%	0.00%	40.00%	60.00%	**100.00%**
59	Golf facilities and other golf industry leaders and stakeholders should commit and can work together to develop an environment that will inspire and recruit more women and girls to play golf, and retain their participation in the game.	0.00%	0.00%	4.00%	28.00%	68.00%	**96.00%**
60	Golf facilities and the golf industry should encourage effective learning and coaching environments, and support entry level play, building on existing initiatives.	0.00%	0.00%	4.00%	40.00%	56.00%	**96.00%**
61	Golf facilities should consider the preferences of the average golfer when setting up the golf course, e.g. length of holes and course, depth and nature of rough, severity of hazards, hole positions, and where necessary make adjustments.	0.00%	0.00%	20.00%	32.00%	48.00%	**80.00%**
62	Facilities should make every effort to promote equality and diversity, and make golf accessible.	0.00%	0.00%	0.00%	32.00%	68.00%	**100.00%**
63	Golf facilities where possible should consider being multi-functional (having facilities in addition to golf- for example gym, walking routes or child care) and having diversity of golf facilities.	0.00%	0.00%	12.00%	56.00%	32.00%	**88.00%**
64	Golf facilities and the golf industry should promote practices that enhance sustainability, -maximising opportunities for wildlife conservation, interaction with green space, restricting water, energy and pesticide/chemical use.	0.00%	0.00%	0.00%	48.00%	52.00%	**100.00%**
65	Golf facilities should be encouraged to provide information and facilities to support golfers warming up to play.	0.00%	0.00%	0.00%	56.00%	44.00%	**100.00%**
66	The golf industry/ golf facilities should encourage players to walk the course if able, and avoid mandatory golf cart use at facilities.	0.00%	0.00%	4.00%	36.00%	60.00%	**96.00%**
67	The golf industry/ golf facilities can encourage and facilitate regular physical activity, and other health enhancing behaviours (for example healthy eating).	0.00%	0.00%	4.00%	44.00%	52.00%	**96.00%**
68	The golf industry should educate and protect employees and golfers about the risks of excess sun exposure.	0.00%	0.00%	4.00%	40.00%	56.00%	**96.00%**
69	Golf facilities should stock sun-screen, hats and collared shirts.	0.00%	4.00%	4.00%	56.00%	36.00%	**92.00%**
70	Golf facilities and the golf industry should continue to support Health and Safety regulations, membership of professional organisations, education relating to safe play, and ensure adequate supervision of children	0.00%	0.00%	4.00%	52.00%	44.00%	**96.00%**
71	Golf facilities should consider providing cardio-pulmonary resuscitation (CPR) training to staff, and provide Automatic External Defibrillators.	0.00%	0.00%	8.00%	32.00%	60.00%	**92.00%**
72	Golf carts should be well maintained, with speed limiters, and front wheel brakes.	0.00%	0.00%	8.00%	44.00%	48.00%	**92.00%**
73	Appropriate lightning safety policies and education should be enacted at each facility. Guidance for appropriate action for players should be highlighted by golf facilities, and the golf industry.	0.00%	0.00%	4.00%	28.00%	68.00%	**96.00%**

d. Actions for policy / decision makers (outwith golf sector)

Question No.	Item	Strongly disagree	Disagree	Neither disagree nor agree	Agree	Strongly agree	% Agree/ Strongly Agree
74	The benefits of regular physical activity including playing golf should be communicated and promoted regularly for persons of all ages, genders, and socio-economic backgrounds.	0.00%	0.00%	0.00%	36.00%	64.00%	**100.00%**
75	Cross –sectoral policies should be delivered that support the World Health Organisation Global Action Plan on Physical Activity, and the United Nations Sustainable Development Goals.	0.00%	0.00%	8.00%	44.00%	48.00%	**92.00%**
76	Policy makers can be confident golf can provide health enhancing physical activity to persons of all ages, and genders. Policy documents, frameworks and actions should support this.	0.00%	0.00%	0.00%	40.00%	60.00%	**100.00%**
77	Policy makers should where relevant include golf as a moderate intensity physical activity in policy documents, guidance and recommendation, and encourage participation for persons of all ages and genders.	0.00%	0.00%	0.00%	33.33%	66.67%	**100.00%**
78	Policy should support play by diverse geographical, and socio-economic participants, of all genders, ages and abilities	0.00%	0.00%	0.00%	33.33%	66.67%	**100.00%**
79	Policy documents, frameworks and actions can where relevant usefully acknowledge green space, health and well-being, nature connection, social and community, local and national economic benefits of golf.	0.00%	0.00%	4.00%	32.00%	64.00%	**96.00%**
80	Policy makers should support efforts to encourage spectators to be physically active (for example walking the course) at golf and other sporting events.	0.00%	0.00%	0.00%	48.00%	52.00%	**100.00%**
81	Policies should promote multi-functionality (having facilities in addition to golf) and diversity of facilities where possible, and sustainable practices	0.00%	0.00%	16.00%	40.00%	44.00%	**84.00%**
82	Policy makers should work collaboratively with the golf industry and national associations to promote increased participation in physical activity/ golf, particularly in groups with low levels of physical activity.	0.00%	0.00%	4.00%	40.00%	56.00%	**96.00%**
83	Policy makers, governing bodies and the golf industry can work collaboratively to gain acceptance from the International Paralympic Committee that golf be included in the Paralympics	0.00%	0.00%	8.33%	41.67%	50.00%	**91.67%**
